# Internal jugular vein thrombosis caused by invasive pharyngeal cancer: a case report and literature review

**DOI:** 10.1016/j.bjorl.2024.101425

**Published:** 2024-03-21

**Authors:** Filip Tudor, Andro Košec, Alan Pegan

**Affiliations:** a University Hospital Center Rijeka, Department of Otorhinolaryngology and Head and Neck Surgery, Rijeka, Croatia; b University Hospital Center Sestre Milosrdnice, Department of Otorhinolaryngology and Head and Neck Surgery, Zagreb, Croatia; c University of Zagreb, School of Medicine, Zagreb, Croatia

## Introduction

Internal Jugular Vein (IJV) thrombosis is a potentially life-threating disease and can occur at any anatomical level. Patients with head and neck cancer are at high-risk category for IJV thrombosis, due to one of two reasons: distant malignancy associated with paraneoplastic syndrome presenting with thrombosis, or locally advanced neoplastic disease in the head and neck area.^1^

Thyroid cancer is a well-known cause of jugular vein thrombosis and despite aggressive multimodal treatment, patients with thyroid cancer that caused IJV thrombosis typically have a poor prognosis, with early lung metastases and high mortality.^2^ The occurrence of IJV thrombosis in head and neck Squamous Cell Carcinoma (SCC) that has directly invaded the IJV is extremely rare.^3^

Tumor invasion of the carotid artery is a known poor prognostic factor, included in the T-stage of the TNM classification.^4^ On the other hand, IJV thrombosis is not documented in the TNM classification, but clearly demonstrates the tumor's aggressiveness.

This case report focuses on a connection with locally advanced head and neck disease and its throbogenic potential, leading to IJV thrombosis.

## Case report

A 62-years-old male patient was referred to a tertiary level hospital due to hoarseness lasting for one month. Clinical examination revealed advanced orohypopharyngeal cancer, extending from the inferior pole of the right tonsil, into the piriform sinus, with an immobile right vocal cord. Painless right-sided neck mass extending through level II, III and IV on the right side of the neck, measuring 6 cm. The patient was in poor general health (ASA 3).

**Figure 1:**
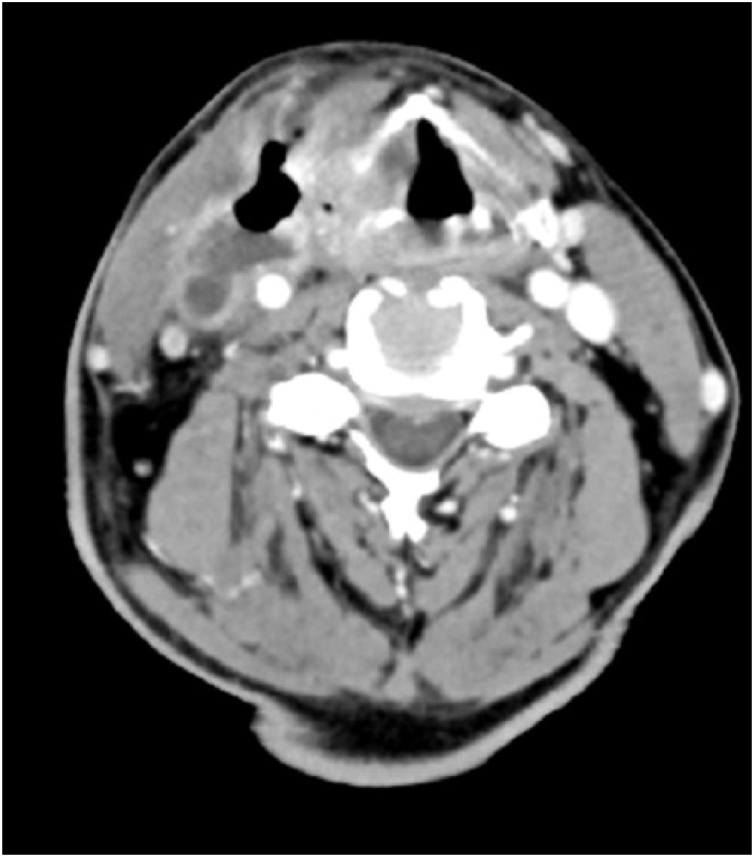
Axial CT scan showing direct spread of tumor through to hypopharyngeal wall and invasion of internal jugular vein.

**Figure 2:**
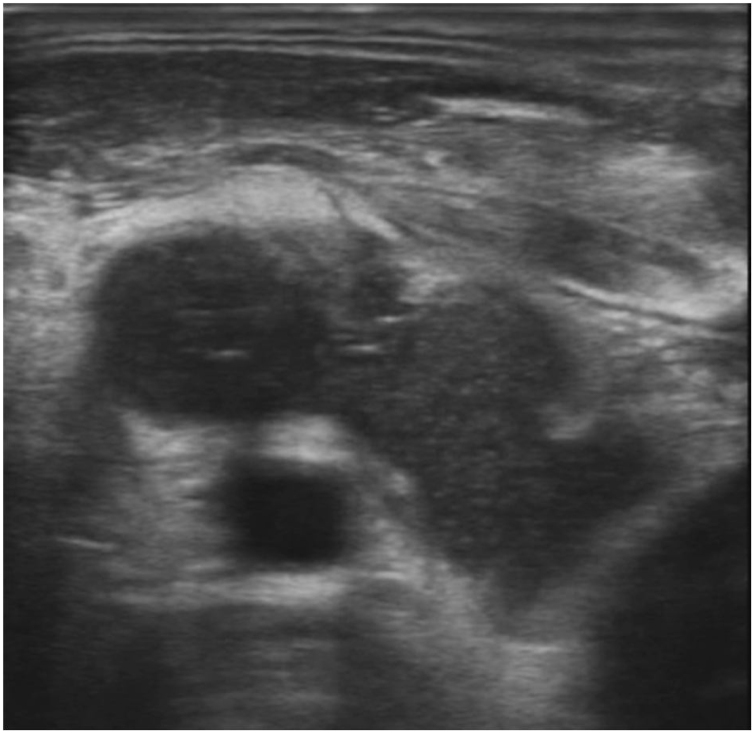
Ultrasonography finding showing right internal jugular vein invasion.

Computer Tomography (CT) of the neck showed a collection of air and fluid (48 × 22 × 65 mm) infiltrating the IJV which was followed continuously from the tumor which infiltrated oropharynx, hypopharynx and larynx. A thrombosed IJV was seen, with thrombus extending from the sigmoid sinus cranially to the subclavian vein caudally. The carotid artery was surrounded up to 180 degrees with the tumor mass, but with maintained blood flow (Fig. 1). Ultrasound examination showed a process measuring 82 × 54 × 33 mm which was infiltrating the IJV, while the common carotid was located posteriorly to the collection (Fig. 2).

Radical surgical treatment was recommended by a multidisciplinary tumor board.

Right sided radical neck dissection and left sided selective neck dissection (levels II‒IV) were performed, followed by total laryngectomy, subtotal pharyngectomy and total thyroidectomy en bloc. Reconstruction was performed with a free fasciocutaneous Anterolateral Thigh Flap (ALT) raised from the right thigh.

**Figure 3:**
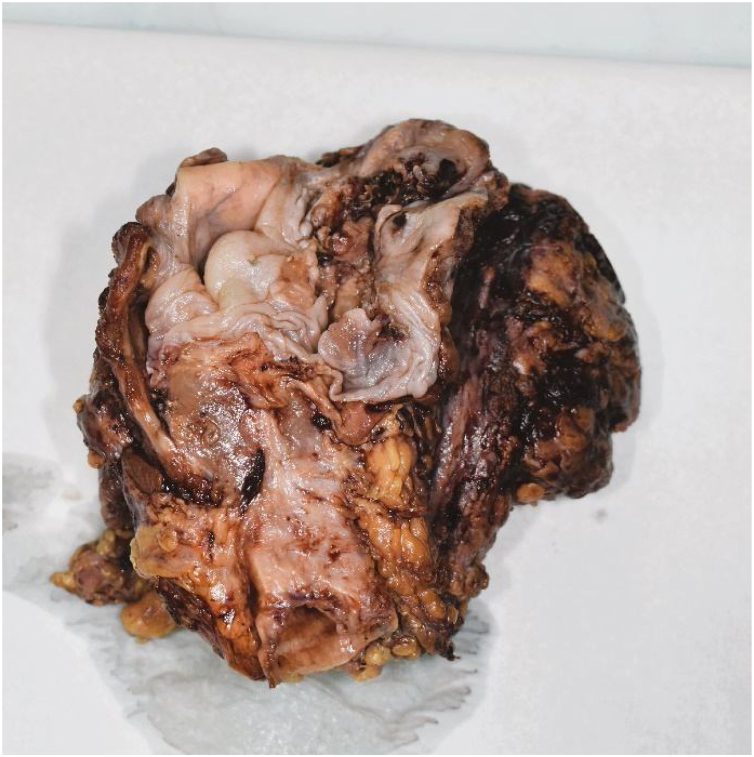
Macroscopic finding of the larynx, pharynx, thyroid gland and right-side neck dissection specimen.

**Figure 4:**
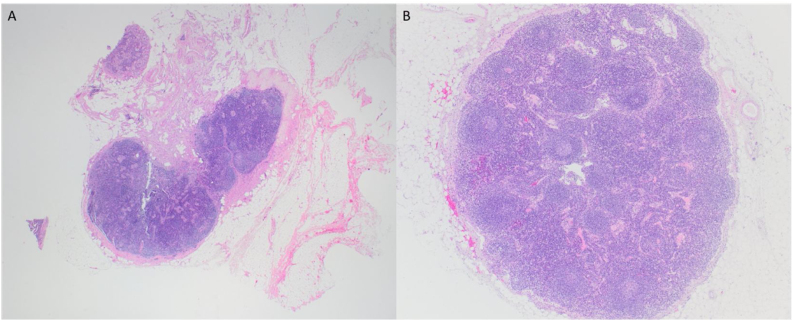
Reactive lymph nodes, 20× magnification (A), 40× magnification (B).

Pathohistological findings confirmed a pT4aN0 moderately differentiated p16-negative SCC with perineural and lymphovascular invasion fully excised with R0 resection margins. Moreover, direct extension of the tumor in the internal jugular vein was seen. Surprisingly, in both of neck dissection specimens, lymph nodes showed reactive changes without tumor infiltration (Figure 3, Figure 4).

Due to poor general condition of the patient and a low performance score, postoperative chemoradiotherapy was not recommended by a multidisciplinary tumor board. Despite the poor prognostic circumstances, the patient is alive, in good general shape, and under regular oncological follow-up 11-months after the surgery.

The associated literature review included a cross-referenced PubMed and Scopus (EMBASE) search using the following keywords and Boolean operators: “head and neck cancer AND squamous cell cancer AND internal jugular vein thrombosis”.

The search resulted with 42 results. Only one publication, which included two case reports, met inclusion criteria.

## Discussion

Only two cases of head and neck SCC causing IJV thrombosis were documented thus far in a single literature report.^5^ The first patient was a 67-year-old male who was treated for T3N3M0 supraglottic SCC. Ultrasonography and CT revealed IJV wall invasion by N3 lymph node causing thrombosis extending to the jugular foramen. He was initially treated with chemoradiotherapy at a total dose of 36 Gy (Cetuximab+1.8 Gy/fraction, 5 fractions/week), and due to progression of the disease, a total pharyngolaryngectomy, bilateral modified radical neck dissection, and jejunal free flap reconstruction were performed. Four months following the surgery patient developed regional recurrence and died 9-months after the surgery.^5^

The second patient was a 65-year-old female who developed T4aN1bM0 cancer of the thyroid gland. MR revealed IJV thrombosis extending from carotid bifurcation to innominate vein bifurcation. The patient was treated with total thyroidectomy, left lateral neck and superior mediastinal dissection. Histopathology revealed SCC with minor component of papillary cancer. Margins were free of tumor. The patient was treated with adjuvant chemoradiotherapy (52 Gy + cisplatin and 5-fluorouracil). Locoregional recurrence was the cause of death 8 months postoperatively.^5^

Thrombosis of internal jugular vein in head and neck squamous cell cancer is extremely rare.^5^ It is assumed that due to the absence of valves and gravity, these vessels are a rare localization of thrombus even in the presence of local malignancies.^3^ In contrast, differentiated thyroid cancer, especially follicular carcinoma, relatively often find a way to cause IJV thrombus.^2^

Due to its low incidence, there are no unanimous recommendations for therapy, while the underlying cause dictates treatment extent and type.^2^ Life-threatening complications have been described after IJV thrombosis, with 10% of patients developing pulmonary embolism.^2^, ^3^ The most common intracranial complications are related with venous ischemia or hemorrhage and include hemiparesis, aphasia and local or general seizures in around 40% of patients with intracranial thrombosis.^3^

It is unclear if primarily oncologic therapy is a better option than surgery, whether surgery should be a cornerstone of therapy, or whether giving supportive care is the best option.

In our case, tumor size and the thrombus that had spread in the cranial part of the internal jugular vein, led us to favor radical surgery as the best option, despite the significant mortality risk. Histopathology did not confirm regional metastatic disease, implying that IJV thrombosis was caused by direct contact with the primary cancer, which has not been documented in the literature prior to this case.

## Conclusion

To the best of our knowledge, this is the first case of IJV thrombosis caused by direct contact with pharyngeal SCC. The surgical details and treatment outcomes described in this case may provide more data on surgical strategies for rare cases where no optimal treatment plan may initially be evident.

## Funding

No funding was received in the preparation of this manuscript.

## Conflicts of interest

The authors declare no onflicts of interest.
